# β-Hydroxybutyrate Inhibits Angiogenesis, Suppresses Non-Small Cell Lung Cancer Growth, and Enhances Gemcitabine Antitumor Activity

**DOI:** 10.3390/ijms27115103

**Published:** 2026-06-04

**Authors:** Yomna Labanie, Kholoud Arafat, Shahrazad Sulaiman, Aya Mudhafar Al-Azawi, Samir Attoub

**Affiliations:** 1 Department of Pharmacology & Therapeutics, College of Medicine & Health Sciences, United Arab Emirates University, Al-Ain 15551, United Arab Emirates; yomna.labanie@uaeu.ac.ae (Y.L.); kholoud.arafat@gmail.com (K.A.); saira992@gmail.com (S.S.); aya.m.alazawi@uaeu.ac.ae (A.M.A.-A.); 2 ASPIRE Precision Medicine Research Institute Abu Dhabi, United Arab Emirates University, Al-Ain 15551, United Arab Emirates

**Keywords:** β-hydroxybutyrate, gemcitabine, NSCLC, colony growth, migration, angiogenesis, tumor growth

## Abstract

Lung cancer remains the most prevalent malignancy and the leading cause of cancer-related mortality worldwide, highlighting the need for novel therapeutic strategies. β-Hydroxybutyrate (BHB), the primary circulating ketone body, has shown antitumor activity in other cancers, but its role in non-small cell lung cancer (NSCLC) is not well defined. This study investigated the anticancer and anti-angiogenic effects of BHB alone and in combination with Gemcitabine using in vitro and in vivo approaches. In A549 (adenocarcinoma) and LNM35 (large-cell carcinoma) NSCLC cells, BHB significantly reduced cell number and colony growth in a concentration-dependent manner, with LNM35 cells exhibiting greater sensitivity. Migration of both A549 and LNM35 cells was also markedly inhibited. In endothelial Telo-HAEC cells, BHB exhibited no cytotoxicity but significantly inhibited migration, tube formation, and VEGF-induced spheroid sprouting, indicating anti-angiogenic activity. Combination studies showed that BHB enhanced Gemcitabine-mediated suppression of NSCLC cell number and colony growth, consistent with an additive to synergistic effect. In the chick embryo chorioallantoic membrane model, BHB and Gemcitabine reduced tumor growth individually, while their combination further decreased tumor weight, particularly in LNM35 xenografts, without observable toxicity. These findings provide preclinical evidence that BHB enhances Gemcitabine antitumor activity against NSCLC and reveals its in vitro anti-angiogenic effects.

## 1. Introduction

According to the latest statistical reports issued by the International Agency for Research on Cancer (IARC), in 2022, lung cancer became the most prevalent cancer worldwide, with 2.5 million new cases, accounting for 12.4% of the total new cancer cases [1]. Despite the continuous efforts and the significant achievements in the development of new lung cancer therapies over the past decade, it remains the primary cause of cancer-related mortality, with 1.8 million deaths, accounting for 18% of the total cancer deaths [1,2]. Moreover, the similarity of symptoms between lung cancer and other more common respiratory diseases, in addition to its nonspecific symptoms, delays its diagnosis in up to 53% of cases. Consequently, this delay reduces the 5-year relative survival rate to 8.9% [3,4,5].

Lung cancer is histologically subdivided into small-cell lung cancer (SCLC) and non-small-cell lung cancer (NSCLC). SCLC includes oat cell carcinoma and combined SCLC, while NSCLC, the most common subtype, includes adenocarcinoma, squamous cell carcinoma, and large cell carcinoma [6,7]. This histological variation reflects the distinct characterization of the molecular profiles and pathways of the different subtypes, underscoring the necessity for specified treatment approaches for the different subtypes of lung cancer [8]. Additionally, resistance, toxicity, and high cost associated with current NSCLC therapies necessitate the development of new alternative or adjuvant treatments [9]. Among these therapies, angiogenesis inhibitors have played an integral role in NSCLC management, and their addition to chemotherapy regimens has resulted in modest improvements in overall survival. Although the clinical interest in these agents has declined due to their toxicities and the emergence of promising checkpoint inhibitors, angiogenesis remains a critical therapeutic target, and there is still a need for safer anti-angiogenic agents in the era of targeted and immunotherapy-based treatments [10].

The ketogenic diet, a high-fat, low-carbohydrate, and adequate-protein regimen [11], works by depleting the body’s glucose stores through carbohydrate restriction [12]. This induces a metabolic shift toward ketogenesis, promoting fatty-acid oxidation in the liver and the production of ketone bodies that serve as an alternative energy source to glucose [13]. Consequently, the ketogenic diet has been proposed as a metabolic intervention that targets the altered energy metabolism characteristic of many cancers. Studies reported that the ketogenic diet may restrict tumor growth, protect normal tissues from collateral damage of anticancer approaches, and enhance tumor sensitivity to chemotherapeutic agents. Its anticancer effect has been explained by the accumulation of ketone bodies [11]. Ketogenesis is initiated in glucose-restricted conditions, where hepatic mitochondria convert free fatty acids into acetyl-CoA through β-oxidation [14]. When acetyl-CoA exceeds the capacity of the tricarboxylic acid (TCA) cycle, it is diverted into ketone synthesis via thiolase and HMG-CoA synthase, forming β-hydroxy-β-methyl glutaryl-CoA (HMG-CoA). HMG-CoA lyase then cleaves this intermediate to yield acetoacetate, which is subsequently reduced by β-hydroxybutyrate dehydrogenase to generate β-hydroxybutyrate (BHB) [13].

BHB is the most prevalent ketone body in mammals. It is a small, polar molecule synthesized by the liver from circulating fatty acids. BHB is converted into acetyl-CoA and subsequently ATP [14], supplying the body with energy during fasting, prolonged exercise, and carbohydrate restriction. BHB also functions as a signaling molecule at the cell surface and intracellularly. It can regulate gene expression, metabolic rate, lipid metabolism, and neuronal function [14]. Studies have demonstrated that BHB can regulate gene expression through various mechanisms, including its inhibitory effect on class I histone deacetylases (HDACs) and its ability to modify histones post-translationally via lysine β-hydroxybutyrylation [K(BHB)] [14]. These properties, particularly its HDAC inhibitory effect, suggest a potential anticancer role for BHB [15].

It has been reported that short-term treatment of NSCLC A549 cells with physiological concentrations of BHB did not significantly impact their viability or migration. However, prolonged treatment resulted in a modest reduction in colony growth compared to the untreated controls [16]. Moreover, in vivo studies demonstrated that 500 mg/Kg of BHB inhibited A549 tumor growth in mice by 43.58% over 13 days of treatment, inducing necrotic damage in tumor tissues without affecting normal tissues [17]. Despite the growing evidence supporting the role of BHB in cancer, its effects on lung cancer remain insufficiently explored. In addition, the impact of BHB on endothelial cells such as TeloHAEC and its combined effect with Gemcitabine, a standard treatment for NSCLC, has not yet been investigated.

We hypothesized that BHB modulates multiple hallmarks of NSCLC, both as a single agent and in combination with standard NSCLC treatments. Accordingly, we investigated the antitumor effects of BHB on NSCLC cell number, colony growth, and migration in vitro using A549 and LNM35 cell lines. In addition, we explored the anti-angiogenic effects of BHB in vitro using TeloHAEC endothelial cells. The in vivo antitumor activity of BHB was further assessed using the chick embryo chorioallantoic membrane (CAM) tumor growth assay, and its combination with Gemcitabine was evaluated in vitro through cell number and colony growth assays, as well as in vivo using the CAM model.

## 2. Results

### 2.1. Effect of BHB on NSCLC Cell Number and Colony Growth In Vitro

**Figure 1:**
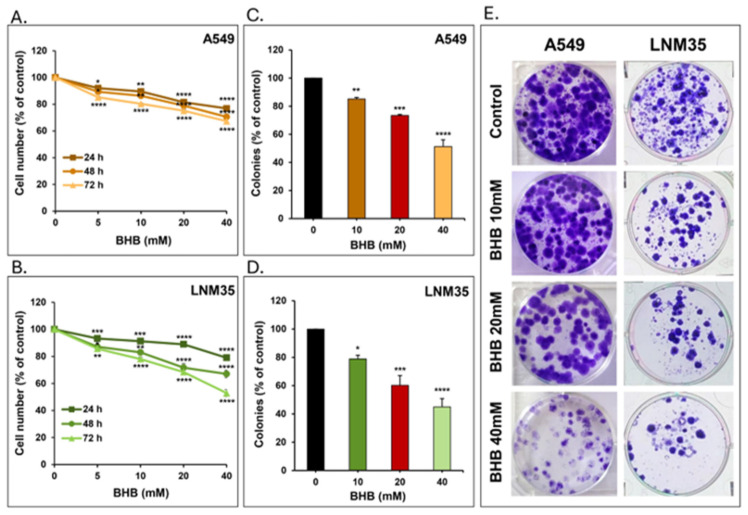
Effect of BHB on NSCLC Cell Number and Colony Growth In vitro. Exponentially growing (**A**) A549 and (**B**) LNM35 cells were incubated in the presence or absence of increasing concentrations of BHB (5 to 40 mM), and cell numbers were determined after 24, 48, and 72 h. (**C**) A549 and (**D**) LNM35 cells were grown for 7 days to form colonies, which were treated with increasing BHB concentrations (10–40 mM) for 14 and 10 days, respectively. Colonies were quantified for A549 and LNM35, respectively. (**E**) Representative pictures of control and BHB-treated colonies for A549 and LNM35 cell lines. Results are expressed as the percentage of treated cells or colonies compared to control cells or colonies. Experiments were performed at least three times. Columns or Shapes represent means. Bars represent S.E.M. Statistical significance was determined using one-way ANOVA followed by Dunnett’s multiple comparison test. (*) Significantly different at *p* < 0.05. (**) Significantly different at *p* < 0.01. (***) Significantly different at *p* < 0.001. (****) Significantly different at *p* < 0.0001.

Escalating concentrations of BHB (5, 10, 20, and 40 mM) reduced the cell number of the NSCLC cell lines A549 and LNM35 in a concentration-dependent manner (Figure 1A,B). To further assess the anticancer potential of BHB in A549 and LNM35 cells, its effect on the growth of formed colonies was evaluated. As shown in Figure 1C–E, BHB treatment induced a concentration-dependent reduction in the total number of colonies in both cell lines. Notably, LNM35 colonies exhibited greater sensitivity to BHB treatment (Figure 1D,E) compared with A549 colonies (Figure 1C,E). Together, these findings confirm the in vitro anticancer efficacy of BHB.

### 2.2. Effect of BHB on NSCLC Cell Migration In Vitro

**Figure 2:**
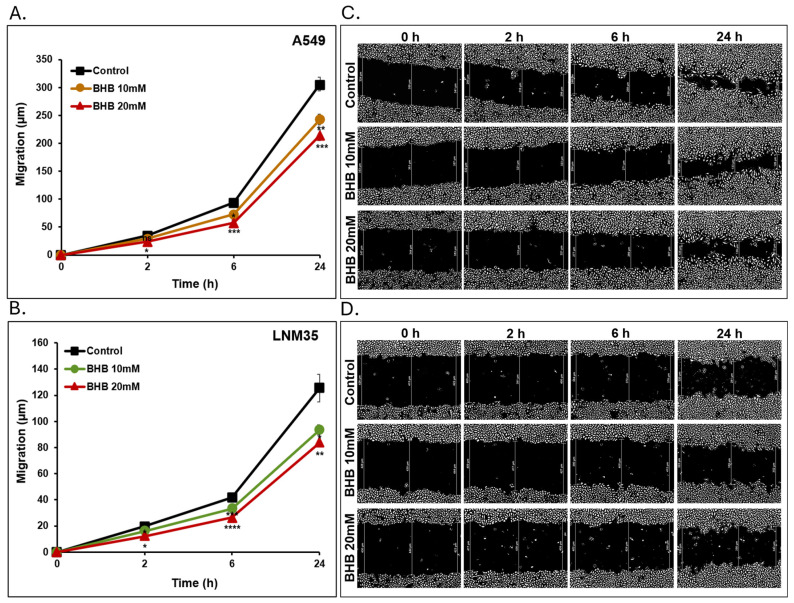
Effect of BHB on NSCLC Cell Migration In vitro using the Wound-Healing Migration Assay. (**A**) A549 and (**B**) LNM35 cells were incubated for 24 h. (**C**,**D**) Photos of induced scratches in the confluent monolayers of A549 and LNM35, respectively, in the presence or absence of BHB (10 and 20 mM) at 0, 2, 6, and 24 h. The scratch width was measured using an inverted microscope with 4× magnification. Measurement was done between the edges of the scraped area. Experiments were performed at least three times. Shapes represent means. Bars represent S.E.M. Statistical significance was determined using one-way ANOVA followed by Dunnett’s multiple comparison test. (ns) Not significant. (*) Significantly different at *p* < 0.05. (**) Significantly different at *p* < 0.01. (***) Significantly different at *p* < 0.001. (****) Significantly different at *p* < 0.0001.

During the transformation into malignancy, tumor cells acquire the ability to migrate and invade the surrounding tissues, initiating metastasis [18]. BHB migration inhibition potential on A549 and LNM35 cells was examined using the wound-healing migration assay. Figure 2 shows the inhibitory effect of BHB on A549 (Figure 2A) and LNM35 (Figure 2B) cells’ migration in a time and concentration-dependent manner. Images of the scratch in different treatment conditions of A549 (Figure 2C) and LNM35 (Figure 2D) were captured at 0, 2, 6, and 24 h.

### 2.3. Effect of BHB on TeloHAEC Cell Migration, Tube Formation, and Sprouting

Cancer cells maintain their essential nutrients and oxygen requirements through angiogenesis, enabling their proliferation and metastasis. Angiogenesis refers to the formation of new blood vessels from existing ones, resulting in a mature functional vascular network. This process entails degrading the basement membrane and extracellular matrix components, activating endothelial cells (ECs), and promoting their proliferation, differentiation, and migration [19,20].

#### 2.3.1. Wound-Healing Migration Assay In Vitro

**Figure 3:**
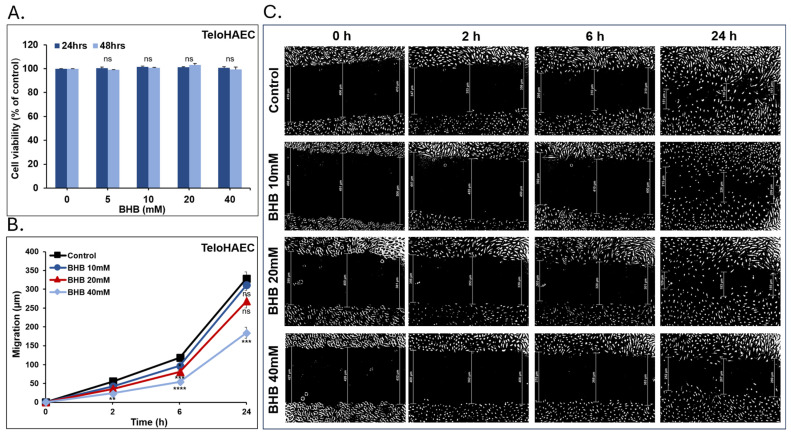
(**A**) Cell viability of TeloHAEC cells incubated with increasing concentrations of BHB (5, 10, 20, and 40 mM) for 24 and 48 h. (**B**) Migration of TeloHAEC cells incubated in the presence or absence of BHB (10, 20, and 40 mM) for 2, 6, and 24 h. (**C**) Representative images of induced scratches in confluent TeloHAEC monolayers captured at 0, 2, 6, and 24 h in the presence or absence of BHB. The scratch width was measured using an inverted microscope with 4× magnification. Measurement was done between the edges of the scraped area. Experiments were performed at least three times. Columns or Shapes represent means. Bars represent S.E.M. Statistical significance was determined using one-way ANOVA followed by Dunnett’s multiple comparison test. (ns) Not significant. (*) Significantly different at *p* < 0.05. (**) Significantly different at *p* < 0.01. (***) Significantly different at *p* < 0.001. (****) Significantly different at *p* < 0.0001.

Treatment with increasing concentrations of BHB (5–40 mM) for 24 and 48 h did not affect the viability of TeloHAEC cells (Figure 3A). The effect of BHB on TeloHAEC cell migration was subsequently evaluated using a wound-healing assay. As shown in Figure 3B, BHB significantly inhibited TeloHAEC migration in a time- and concentration-dependent manner at 2, 6, and 24 h. Representative images of the wound area under the different treatment conditions are shown in Figure 3C and were captured at 0, 2, 6, and 24 h.

#### 2.3.2. Capillary-like Tube Formation Assay In Vitro

**Figure 4:**
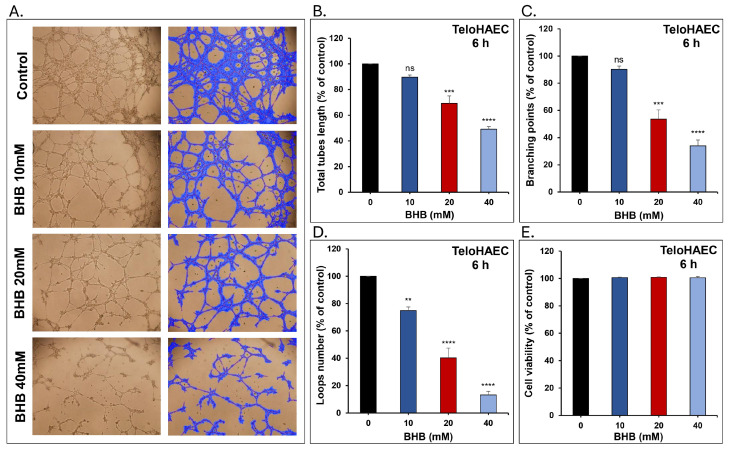
Effect of BHB on TeloHAEC Cell Capillary-like Tube Formation In vitro. TeloHAEC cells were cultured on a Matrigel matrix in the presence or absence of different BHB concentrations (10, 20, and 40 mM) for 6 h. (**A**) Photos are captured using an inverted microscope (4× times 1.6) and analyzed for different quantification parameters: (**B**) total tubes’ length formed, (**C**) total branching points, and (**D**) total loop number using Wimasis software. (**E**) Cell viability. Experiments were performed at least three times. Columns are means. Bars are S.E.M. Statistical significance was determined using one-way ANOVA followed by Dunnett’s multiple comparison test. (ns) Not significant. (**) Significantly different at *p* < 0.01. (***) Significantly different at *p* < 0.001. (****) Significantly different at *p* < 0.0001.

The anti-angiogenic effect of BHB was investigated using TeloHAEC cells in the in vitro capillary-like tube formation assay. As shown in Figure 4A, in the absence of BHB, TeloHAEC cells form an organized capillary-like network. Treating TeloHAEC cells with increasing BHB concentrations disrupts this organized network in a concentration-dependent manner. BHB causes a gradual reduction in the total length of formed tubes (Figure 4B), the total number of branching points (Figure 4C), and the total number of loops formed (Figure 4D). 20 mM of BHB was able to inhibit the formation of the capillary-like structure by 31%, branching points by 46%, and loop number by 60%. Cell viability was measured in these conditions at the end of the experiment, indicating that the antiangiogenic effect of BHB was not caused by a reduction in cell viability (Figure 4E).

#### 2.3.3. Transwell Migration Assay and Spheroids’ Sprouts Formation In Vitro

**Figure 5:**
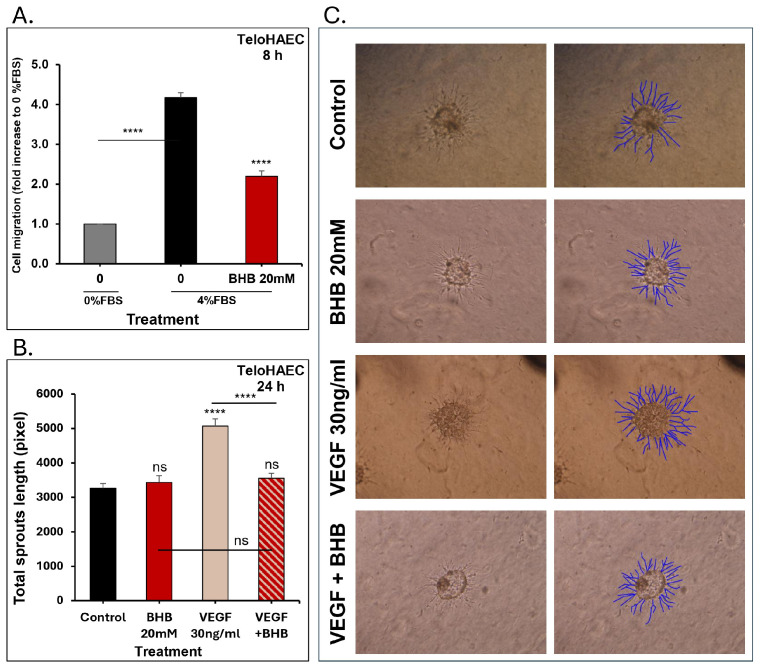
Effect of BHB on TeloHAEC Cell Migration and Spheroids’ Sprouts Formation In vitro. (**A**) In the transwell migration assay, TeloHAEC cells were incubated with or without 20 mM of BHB for 8 h against 0 or 4% FBS-containing medium. Cells that migrated through the 8 μm pores were determined using the cell viability assay. (**B**) Collagen-embedded TeloHAEC spheroids were incubated for 24 h with or without 20 mM BHB, 30 ng/mL VEGF, or their combination, then total sprout length was quantified, and the average sprout length was calculated for each condition. (**C**) An inverted microscope at (20×) was used to capture the spheroids with formed sprouts’ photos. The total sprout length was quantified using ImageJ software (version 1.54g built on the Java 1.8.0_345 platform). The Experiment was performed three times. Sprout formation data are from a representative experiment. Columns are means. Bars are S.E.M. For panel (**A**), statistical significance was determined using one-way ANOVA followed by Dunnett’s multiple comparison test. For panel (**B**), statistical significance was determined using one-way ANOVA followed by Tukey’s multiple comparison test. (ns) Not significant. (****) Significantly different at *p* < 0.0001.

Since cell migration is part of the sprouting process, the transwell migration assay was first used to confirm the effect of BHB on TeloHAEC cell migration. We decided to use 20 mM of BHB as it was found to be the lowest effective concentration shown in previous experiments. As shown in Figure 5A, 4% FBS-containing medium stimulated the migration of TeloHAEC cells through the insert pores from the upper to the lower chamber. The increase in cell migration was 3.2 times more than the migration of the TeloHAEC cells stimulated by 0% FBS-containing medium. Treating TeloHAEC cells with 20 mM of BHB induced approximately a 50% reduction in cell migration stimulated by the 4% FBS-containing media.

Secondly, the anti-angiogenic effect of 20 mM BHB was investigated in a 3D spheroid sprouting model. TeloHAEC spheroids were embedded in a collagen matrix in the presence or absence of 30 ng/mL VEGF, 20 mM BHB, or their combination. As observed in (Figure 5B,C), 30 ng/mL VEGF induced a significant increase in the total sprouts’ length. Treating spheroids with 20 mM BHB did not affect the total basal sprouts’ length; however, when BHB was combined with VEGF, it completely reversed the VEGF sprouting stimulatory effect.

### 2.4. Effect of BHB, Gemcitabine, and Their Combination on the NSCLC Cell Number, Colony, and Tumor Growth

Combining therapeutic agents that act through different molecular mechanisms has been shown to enhance efficacy compared to individual treatments, while also reducing side effects and the development of drug resistance [21]. Gemcitabine, an antimetabolite chemotherapeutic agent, is commonly used in combination with Cisplatin for the treatment of stage IIIA, IIIB, or stage IV NSCLC [22]. Accordingly, we investigated the combined effects of BHB and Gemcitabine on NSCLC cell lines and tumor growth.

#### 2.4.1. Cell Number Assay In Vitro

**Figure 6:**
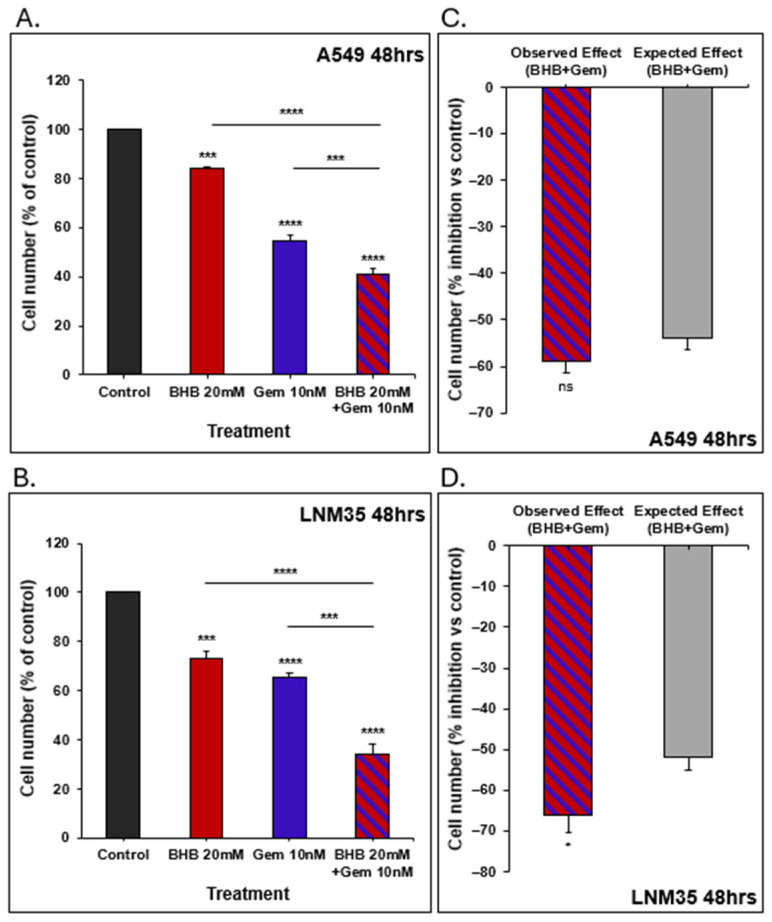
(**A**) A549 and (**B**) LNM35 cells were incubated in the presence or absence of BHB (20 mM), Gemcitabine (10 nM), or their combination for 48 h. (**C**,**D**) Comparison of the observed combination effect on % cell number inhibition in A549 and LNM35 cells, respectively, with the expected combination effect calculated using the Bliss independence model. Experiments were performed at least three times. Columns are means. Bars are S.E.M. Statistical significance was determined using one-way ANOVA followed by Tukey’s multiple comparison test. (ns) Not significant. (*) Significantly different at *p* < 0.05. (***) Significantly different at *p* < 0.001. (****) Significantly different at *p* < 0.0001.

The effects of BHB, Gemcitabine, and their combination were evaluated on A549 and LNM35 cell lines. Cells were treated with 20 mM BHB and 10 nM Gemcitabine for 48 h. As shown in Figure 6A and B, the combination treatment produced a greater reduction in cell number compared with either agent alone. The Bliss independence model was applied to evaluate the type of pharmacological interaction. Based on the EOB calculation and the comparison between observed and expected combination effects, an additive effect was observed in A549 cells (Figure 6C), whereas a synergistic effect was detected in LNM35 cells (Figure 6D).

#### 2.4.2. Colony Growth Assay In Vitro

**Figure 7:**
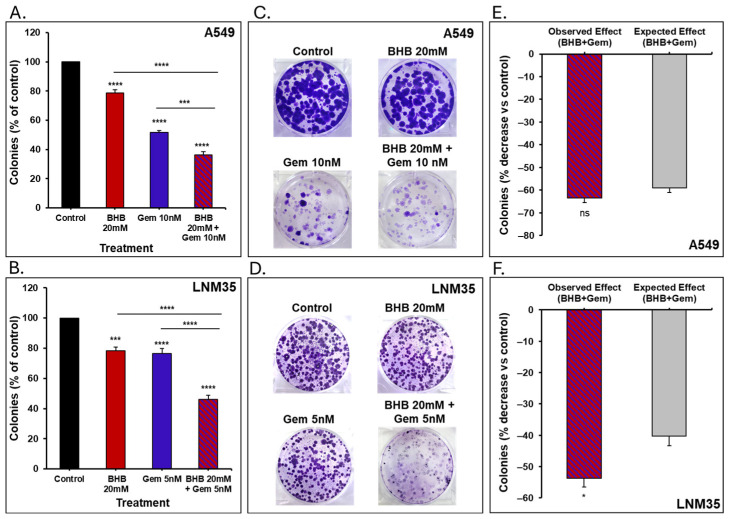
Effect of BHB, Gemcitabine, and their Combination on Colony Growth of NSCLC Cell Lines In vitro. (**A**) A549 cells were grown for 7 days to form colonies, then treated with 20 mM BHB, 10 nM Gemcitabine, or their combination for 14 days. (**B**) LNM35 cells were grown for 7 days to form colonies, then treated with 20 mM BHB, 5 nM Gemcitabine, or their combination for 10 days. (**A**,**B**) Colonies count in A549 and LNM35, respectively. (**C**,**D**) Representative pictures of control and treated colonies for A549 and LNM35 cell lines, respectively. (**E**,**F**) Comparison of the expected combination effect, calculated using the Bliss independence model, with the observed combination effect on A549 and LNM35 colonies, respectively. Experiments were performed at least three times. Results are expressed as the percentage of treated colonies compared to the control. Columns represent means. Bars represent S.E.M. For panels (**A**,**B**), statistical significance was determined using one-way ANOVA followed by Tukey’s multiple comparison test. While for panels (**E**,**F**), statistical significance was determined using an unpaired *t*-test. (ns) Not significant. (*) Significantly different at *p* < 0.05. (***) Significantly different at *p* < 0.001. (****) Significantly different at *p* < 0.0001.

We further investigated the anticancer effects of the combined BHB and Gemcitabine treatment on the growth of pre-formed colonies of A549 and LNM35 cells. A549 colonies were treated for 14 days, whereas LNM35 colonies were treated for 10 days. As shown in Figure 7A–D, the combination treatment resulted in greater inhibition of colony growth compared with either treatment alone. Consistent with the cell proliferation assay, the combination treatment exhibited an additive effect in A549 colonies (Figure 7E) and a synergistic effect in LNM35 colonies (Figure 7F).

#### 2.4.3. Tumor Growth CAM Assay In Vivo

**Figure 8:**
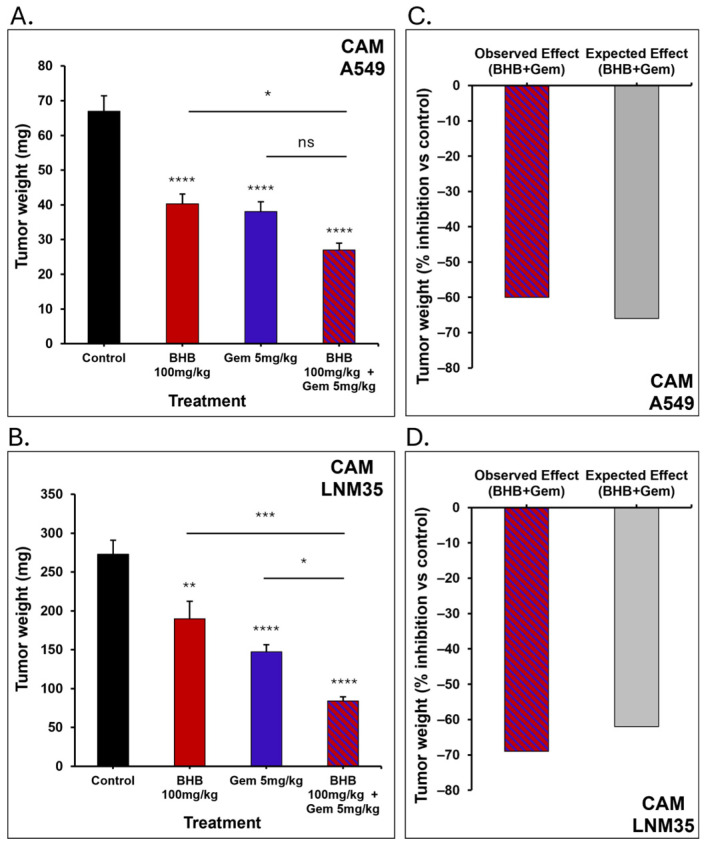
Effect of BHB, Gemcitabine, and their Combination on A549 and LNM35 Tumor Growth In vivo using CAM Assay. Tumor weight of A549 (**A**) and LNM35 (**B**) xenografts grown on the chick embryo CAM following treatment with vehicle, BHB (100 mg/kg), Gemcitabine (5 mg/kg), or their combination. Treatments were administered on alternate days for a total of three treatments. (**C**,**D**) Comparison of the expected combination effect, calculated using the Bliss independence model, with the observed combination effect on A549 and LNM35 tumor weight, respectively. Columns are means. Bars are S.E.M. Statistical significance was determined using one-way ANOVA followed by Tukey’s multiple comparison test. (ns) Not significant. (*) Significantly different at *p* < 0.05. (**) Significantly different at *p* < 0.01. (***) Significantly different at *p* < 0.001. (****) Significantly different at *p* < 0.0001.

The impact of BHB, Gemcitabine, and their combination on the NSCLC tumors was investigated in vivo using the chick embryo CAM assay. Figure 8A shows that treating A549 tumors with 100 mg/Kg BHB and 5 mg/Kg Gemcitabine resulted in a 40% and 43% reduction in tumor weight, respectively, compared to the control, while the combination treatment improved the inhibitory effect by 20% compared to BHB and 17% compared to Gemcitabine. The 60% reduction in tumor weight observed with the combination treatment was close to the expected combination effect of 66% relative to the control group (Figure 8C). On the other hand, as observed in Figure 8B, LNM35 tumors treated with 100 mg/Kg BHB and 5 mg/Kg Gemcitabine showed a 30% and 46% reduction in tumor weight, respectively, compared to the control. Remarkably, the administration of 100 mg/kg BHB and 5 mg/kg Gemcitabine combination enhanced the inhibitory effect on LNM35 tumors by 39% compared to BHB and 23% compared to Gemcitabine. The 69% reduction in tumor weight observed with the combination treatment was close to the expected combination effect of 62% relative to the control group (Figure 8D).

The toxicity of the individual and combination treatments was evaluated by pooling and comparing the survival rates of chick embryos xenografted with either A549 or LNM35 tumors. At E17, 96.6% of embryos in the control groups remained viable. Treatment with BHB or Gemcitabine alone resulted in survival rates of 93% and 100%, respectively, indicating no significant toxicity. Importantly, the combination of 100 mg/Kg BHB and 5 mg/Kg Gemcitabine resulted in 89.8% embryo survival at E17, corresponding to only a 6.8% reduction in viability compared with the control group, suggesting limited toxicity of the combination treatment.

## 3. Discussion

Cancer cells were first observed to consume more glucose than normal cells in 1922 when Braunstein noticed the glucose-free urine of diabetic patients diagnosed with cancer. After that, Otto Warburg confirmed this observation. He reported the characteristic glucose consumption and lactate production in cancer cells, even when oxygen is available, the so-called Warburg effect [23]. This phenomenon has been regarded as a cornerstone in metabolic reprogramming [24]. Interestingly, understanding the metabolic alterations provides insights into cancer cells’ nature, aiding in their diagnosis and enabling therapy customization for various cancer types [25].

Braunstein and Warburg’s findings suggested the potential utility of the ketogenic diet as an adjuvant to cancer therapy, targeting cancer cell starvation [26]. Despite the positive outcomes of the ketogenic diet as an adjuvant to cancer therapy [11], several adverse effects, such as nutrient deficiencies, digestive issues, kidney stone formation [26], and excessive weight loss, limit the use of this approach [23,27]. Therefore, current studies are focused on finding alternatives to the strict ketogenic diet by supplementing cancer patients with ketone bodies [27]. It was documented that treatment with the main ketogenic body, BHB, produced potential anti-tumor effects in numerous cancers. Dmitrieva-Posocco et al. reported that BHB effectively reduces the proliferation of colonic crypt cells and significantly suppresses intestinal tumor growth using the AOM/DSS mice colorectal cancer model and HT-29 colorectal cancer cells [28]. Similar antitumor effects of BHB have been observed in various cancer cell lines, including U251 glioma cells [29], LNCaP and PC3 prostate cancer cells [30], and CaKi-1 clear renal carcinoma cells [31]. It was also proven that hydroxy-methyl-glutaryl-CoA lyase (HMGCL)-induced BHB production suppresses Huh7 and MHCC-LM3 hepatocellular carcinoma cells’ proliferation and metastasis via ferroptosis stimulation [32]. In contrast, other cancer types were able to utilize ketone bodies as an energy source during glucose-deprived conditions, like normal cells. For instance, MMTV-NEU-NT mammary tumors [33], SW480 colorectal cancer cells [34], PDA-bearing KIC mice, PICNA-1, and MiaPaCa-2 human pancreatic cancer cells [35], and SW1353 chondrosarcoma cells [36] utilized BHB to fuel their tumorigenesis. With the variable response of BHB reported across the different types of cancer, its impact on NSCLC remains insufficiently explored.

We show that treatment of A549 and LNM35 cells with increasing concentrations of BHB (5 to 40 mM) for 24, 48, and 72 h resulted in a concentration-dependent reduction in cell number, supporting a direct inhibitory effect of BHB on NSCLC cell growth. Notably, LNM35 cells were more sensitive to BHB treatment compared to A549 cells. Our findings for A549 cells complement the previously available data. It was reported that 48 h treatment with BHB (1 μM to 3 mM) did not affect A549 cell viability [16]. Additionally, treatment with BHB (1.56 to 25 mM) for 24 and 48 h showed a significant reduction in its cell viability at concentrations of 12.5 mM or higher [37]. Likewise, treating glioma U251 cells with BHB (0.5 to 8 mM) for 24 h [29], renal carcinoma Caki-1 cells with BHB (1 to 200 mM) for 48 and 72 h [31], and prostate cancer LNCaP, PC3, and DU145 cells with BHB (1 to 24 mM) for 48 h [30] produced a comparable reduction in cell viability. Furthermore, the treatment of HT-29 colorectal cancer (CRC) cells with BHB (10 to 40 mM) for 3 or 6 days resulted in a gradual reduction in cell proliferation [28]. In contrast, treating CRC SW480 cells with BHB (0.1 to 5 mM) for 72 h caused an increase in their cell viability [34]. Moreover, treating the chondrosarcoma SW1353 cells with BHB (10 or 25 mM) for 24 and 48 h, both with or without FBS, either rescued the cells from starvation or enhanced their proliferation [36]. Besides that, BHB (0.5 to 200 mM) treatment is tolerated by breast cancer T47D and MCF-7 cells up to 25 and 50 mM, respectively. It failed to rescue T47D and MCF-7 cells during glucose-deprived conditions [38]. In the case of TeloHAEC endothelial cells, cell viability remained unaffected by BHB with concentrations up to 40 mM for 24 to 48 h, suggesting BHB safety for normal cells.

The current study demonstrates that long-term treatment of A549 (14 days) and LNM35 (10 days) with escalating BHB concentrations (10 to 40 mM) decreased the colony growth in a concentration-dependent manner. Similarly, in line with the cell number assay, LNM35 cells show higher sensitivity to BHB than A549 cells. As demonstrated in a previous study, A549 cells treated for 8 days with 3 mM of BHB exhibited a slight decrease in colony growth compared to untreated cells [16]. This suggests that physiological concentrations of BHB have weak anticancer effects on A549 cells. Previous investigations on different cancer cell lines supported the anticancer effect of BHB. It was reported that treating prostate cancer LNCaP and PC3 colonies with 15 mM BHB for 2 days [30], and glioma U251 colonies with 2 mM of BHB for 10 days [29], suppressed their proliferation capacity, decreasing the number of colonies formed. Conversely, the CRC SW480 colony formation significantly increased compared to the control after treatment with 250 μM of BHB for 14 days [34].

Tumor cells acquire genetic mutations that enable them to migrate and invade surrounding tissues, initiating their metastatic cascade [39]. It was found in a previous investigation that 3 mM of BHB slightly reduced A549 cell migration compared to control in the first 24 h. However, the effect was not evident in the second two days [16]. In our study, we evaluated the effect of BHB on the migration of A549 and LNM35. After BHB treatment (10 and 20 mM) for 24 h, we observed that LNM35 cells were again more sensitive to BHB-induced migration inhibition than A549 cells. Consistent with our findings, a study investigating the effect of BHB on prostate cancer revealed that treating LNCaP and PC3 cells with 15 mM of BHB for 24 h significantly inhibited cell migration [30]. In addition, the cell migration of glioma U251 cells treated with 2 mM of BHB for 24 h was markedly reduced compared to untreated cells [29]. On the other hand, a study noted a significant increase in the cell migration ability of CRC SW480 cells after 36 and 72 h of treatment with 250 μM of BHB [34].

During tumor progression, rapid cell proliferation and intense metabolism increase the demand for oxygen and nutrients. If the surrounding tissue fails to meet these demands, tumor cells release pro-angiogenic factors to stimulate nearby endothelial cell proliferation and migration, forming new blood vessels [40]. In this study, we reported that BHB inhibited TeloHAEC cell migration in a time and concentration-dependent manner. Moreover, we found that BHB was able to inhibit the capillary-like tube formation in a concentration-dependent manner without affecting cell viability during short-term treatment. To further understand the effect of BHB on angiogenesis, we performed a 3D sprouting model treatment. Although 20 mM of BHB did not reduce the total basal sprout length formed by TeloHAEC spheroids, it completely reversed the VEGF sprouting stimulatory effect. The impact of BHB on TeloHAEC tube formation and VEGF-induced sprouts’ inhibition was not reported previously. However, recent investigations reported that 2 and 50 mM of BHB reduced the expression level of VEGF in U251 glioma and Caki-1 renal carcinoma cells, respectively [29,41].

Combining anti-cancer agents is an essential strategy in cancer therapy. Combination therapy enhances the efficacy of the combined agents compared to monotherapy by targeting the main anti-tumor pathways in an additive or synergistic manner. This approach also reduces drug resistance and toxicity associated with individual treatments, as lower doses are administered compared to monotherapy [21]. Gemcitabine, an analogue of deoxycytidine, is a pyrimidine antimetabolite that acts by interfering with DNA synthesis, leading to cell death [42]. The combination of Gemcitabine and cisplatin is used as a first-line therapy for advanced NSCLC and is considered the most cost-effective regimen among platinum-based combinations with third-generation cytotoxic agents [43]. Studies on various cancer models reported that the effectiveness of Gemcitabine is potentiated when administered alongside a ketogenic diet [44,45]. However, the specific metabolite mediating this enhancement has not been clearly identified. In addition, clinical findings indicated that fatty acid supplementation can improve the therapeutic efficacy and tolerability of Gemcitabine [46]. Together, these findings suggest a possible metabolic interaction between Gemcitabine and BHB, thereby providing a strong scientific basis for investigating their combination in NSCLC.

In the present study, we combined BHB with Gemcitabine and tested their effect using in vitro and in vivo assays on A549 and LNM35 cell lines. The combination showed a higher inhibitory effect on cell number compared to individual treatments. Furthermore, it exhibited stronger and more significant inhibition of colony growth in both cell lines. Distinctly, the effect of the combination treatment was more pronounced on LNM35 than on A549 cells in both assays. We validated our in vitro findings by assessing the effects of BHB, alone and in combination with Gemcitabine, on tumor progression in vivo using the chick embryo CAM assay. Topical application of BHB onto the CAM at a dose of 100 mg/kg resulted in a significant reduction in tumor growth of both A549 and LNM35 xenografts, achieving approximately 40% and 30% inhibition, respectively. The CAM dose was selected based on previously in vivo reported tolerability ranges for BHB (100–500 mg/kg) and was further validated in our model by high embryo survival rates and the absence of obvious toxicity, while biological activity was assessed using tumor inhibition as the primary experimental endpoint. While further investigations on CAM were not documented in the literature, the impact of BHB on xenografted tumors was reported in nude mice. It was reported that the administration of 500 mg/Kg of BHB intraperitoneally for 13 successive days was able to reduce A549 tumor growth by around 43.58% [17]. Similarly, daily intraperitoneal administration of 100 mg/Kg of BHB for up to 30 days to LNCaP prostate cancer-bearing mice was able to significantly attenuate tumor growth [30]. We additionally reported that Gemcitabine significantly enhanced the BHB-induced reduction in tumor growth, resulting in a 60% and 69% decrease in tumor weight in A549 and LNM35 xenografts, respectively.

It was interestingly observed that LNM35 is more sensitive to BHB than A549 in most of the experiments done. This variation in response is possibly a result of the difference in their histological origin and genetic profile. A549 cell line represents a lung adenocarcinoma, derived from human type II alveolar epithelial cells [47], whereas LNM35 (a subline of NCI-H460) is a large-cell carcinoma characterized by high tumorigenicity, invasiveness, and strong lymphogenous metastatic potential. LNM35 was identified as the first human lung cancer cell line capable of spontaneous lymph node metastasis when xenografted into nude mice, reflecting its more aggressive phenotype [48]. Additionally, NCI-H460 is known to have a higher glycolysis rate in comparison to A549 due to the differential expression of glycolytic enzymes such as HKII and MCT4 [49,50]. These differences in the metabolic profiles between the two cell lines could partially explain the variation in the response observed in this study and other reports. For instance, Cunha et al. (2022) found that NCI-H460 is more sensitive to glycolysis inhibitors, such as 3-bromopyruvate (3BP), dichloroacetate (DCA), and 2-deoxyglucose (2DG) [49]. Similarly, Al-Azawi et al. (2021) showed that DCA has a higher effect on LNM35 clonogenic growth compared to A549 [51].

Toxicological screening is crucial for both new drug development and the enhancement of existing molecular therapeutic applications [52]. Ensuring the safety of a treatment is as important as its effectiveness [53]. According to our results, BHB, Gemcitabine, and their combination showed no significant toxicity to the chick embryos, with 3.5%, 0%, and 6.9% reduction in survival rate, respectively, compared to the control at the end of the experiment. In a previous preclinical BHB safety study, rats were fed a diet with 11.4% ketone monoester (94% (R)-3-hydroxybutyl (R)-3-hydroxybutyrate) for 28 days; results showed no adverse effects. All animals survived until the scheduled necropsy date, and physical examinations indicated no treatment-related toxicity [54]. Clinically, it was reported that a daily intake of 25.5 and 7.5 g of BHB for 90 days was safe for healthy adults and adolescents, respectively. It showed no adverse effects on blood health markers, psychological well-being, or cardiovascular markers [55,56].

Although elucidating the mechanistic actions of BHB was not within the primary scope of our investigation, we consider it important to highlight several potential mechanisms through which BHB may exert its effects. BHB is known to signal through the hydroxycarboxylic acid receptor 2 (HCAR2/GPR109A), a Gi-coupled GPCR, which has been implicated in the suppression of pro-inflammatory and pro-angiogenic signaling [57]. This mechanism is consistent with the anti-migratory and anti-angiogenic effects we observed in TeloHAEC cells. Furthermore, BHB has been associated with alterations in cellular energy metabolism and redox status, including potential activation of AMPK and modulation of Akt/mTOR signaling [57,58]. Such effects could attenuate anabolic and survival pathways while sensitizing NSCLC cells to Gemcitabine, in line with the enhanced reduction in cell number, colony, and tumor growth seen with the combination treatment. Additionally, BHB inhibits the expression of glycolytic enzymes, such as PFKFB3 and GLUT1, in hypoxic cardiomyocytes [59]. Since glycolysis plays an integral role in the proliferating endothelial cells [60], it is speculated that BHB inhibits angiogenesis by suppressing glycolysis, a mechanism that warrants further investigation.

In summary, our study highlights the anti-cancer potential of BHB against NSCLC, demonstrating its inhibitory effects on cell number, colony growth, and migration in vitro, as well as tumor growth in vivo using the chick embryo CAM model, while maintaining a favorable safety profile. Furthermore, BHB exhibited significant antiangiogenic activity by suppressing endothelial cell migration, capillary-like tube formation, and sprouting in vitro. Lastly, we established the efficacy of BHB in combination with Gemcitabine, enhancing its impact on cell proliferation, colony growth in vitro, and tumor growth inhibition in vivo.

## 4. Materials and Methods

### 4.1. Cell Culture and Reagents

Human NSCLC, A549 cells were procured from the American Type Culture Collection (ATCC, Manassas, VA, USA), whereas LNM35 cells were provided generously by Prof. Takahashi. Both cellular models, A549 and LNM35, were maintained and cultured in RPMI-1640 medium (Gibco, Paisley, UK). hTERT-immortalized human aortic endothelial cells (TeloHAEC) were maintained in an EMB-2 media kit (CC3162 Lonza, Walkersville, MD, USA). All cancer cell media were supplemented with Antibiotic-Antimycotic consisting of 10,000 units/mL of penicillin, 10,000 μg/mL of streptomycin, and 25 μg/mL of Gibco Amphotericin B (Gibco, Grand Island, NY, USA) and 10% fetal bovine serum (Gibco, Paisley, UK). All cell lines were incubated in a humidified incubator at 37 °C and 5% CO_2_. The cells’ culturing media were replaced every three days, and the cells were passed once weekly, upon reaching 95% confluence.

β-Hydroxybutyrate (in the form of (±)-Sodium 3-hydroxybutyrate; Ref: 54965-50G-F) and Gemcitabine (in the form of Gemcitabine hydrochloride, Ref: G6423-10MG) were purchased from Sigma-Aldrich (St. Louis, MO, USA). Before each experiment, BHB was freshly prepared and diluted to the required concentrations using the appropriate culture medium specific to each cell line.

### 4.2. Cell Number Assay

A549 and LNM35 cells were seeded at equal densities (80,000 c/w) in 12-well plates. After 24 h of incubation, cells were treated with an increasing concentration of BHB (5, 10, 20, and 40 mM) in duplicates for 24, 48, and 72 h, while control wells were treated with equal amounts of medium at the specified time intervals. The BHB effect on the cell number of the tested cells was determined using the CellDrop Automated cell counter (DeNovix Inc., Wilmington, DE, USA). In the second set of experiments, cells were treated with 20 mM of BHB, 10 nM of Gemcitabine, or their combination for 48 h. Cell number was expressed as a percentage (%) by comparing the number of treated cells to that of control cells, which were assumed to be 100%.

### 4.3. Cell Viability Assay

TeloHAEC cell line was seeded in 96-well plates at a density of 9000 cells per well. After 24 h of incubation, cells were treated with an increasing concentration of BHB (5, 10, 20, and 40 mM) in duplicates for 24 and 48 h, while control wells were treated with equal amounts of medium. BHB effect on the cell viability of the tested cell line was determined using the CellTiter-Glo^®^ Luminescent Cell Viability assay from Promega Corporation (Madison, WI, USA), quantifying ATP. The measured ATP in each condition reflects the metabolically active cells. GloMax^®^ Luminometer (Promega Corporation, Madison, WI, USA) was used to quantify the luminescent signal produced. Cell viability was expressed as a percentage (%) by comparing the viability of BHB-treated cells to that of control cells, which were assumed to be 100% viable.

### 4.4. Colony Growth Assay

A549 and LMN35 cells were seeded in 6-well plates at densities of 50 and 100 cells/well, respectively. Plates were then kept in a humidified incubator at 37 °C and 5% CO_2_ for 7 days, allowing colony formation. Formed colonies were treated in duplicates with increasing concentrations of BHB (10, 20, and 40 mM), every 3 days, up to 10 days for the LNM35 cell line, and up to 14 days for the A549 cell line. Control wells were treated with a similar volume of medium.

On the stopping day, colonies were fixed and stained using 0.5% crystal violet dissolved in 50% methanol (*v*/*v*) for 30 min. Thereafter, colonies were washed with tap water, and air drying was allowed. Colonies containing 50 or more cells were enumerated using an inverted microscope (Olympus I×71, Olympus Corporation, Tokyo, Japan), with those containing over 200 cells classified as large colonies. A second set of experiments was done using the same procedure; however, the formed colonies were treated with BHB, Gemcitabine, or their combination. The experimental data were represented as colony percentage (%) by comparing drug-treated colonies to control colonies, which were considered as 100%.

### 4.5. Wound Healing Migration Assay

A549, LNM35, and TeloHAEC cells were seeded in 12-well plates and incubated for 24 h to allow the formation of a confluent monolayer. Using a 200 μL pipette tip, a scratch was made in the center of each well, with subsequent cell washing twice using 1× DPBS. Afterward, control wells were supplemented with fresh medium, whereas treated wells were supplemented with medium containing different BHB concentrations in duplicates. Using an inverted microscope at a 4× objective lens magnification (Olympus I×71, Olympus Corporation, Tokyo, Japan), part of the scratch from each well was chosen to be examined, and its location was marked on the plate cover. Images were captured, and the scratch widths were measured at 0, 2, 6, and 24 h of treatment. The migration distance was calculated as the mean of the difference between the measurements at the indicated time points.

### 4.6. Transwell Migration Assay

The impact of BHB on TeloHAEC endothelial cells’ migration was tested using the transwell migration kit (Corning Incorporated, Corning, NY, USA). In this set of experiments, two control conditions were used. In the first control, 5 × 10^4^ cells were seeded in 0.5 mL of 0.1% FBS-supplemented medium in the upper chamber and 0.75 mL of 0% FBS-supplemented medium in the lower chamber. In the second control, 5 × 10^4^ cells were seeded in 0.5 mL of 0.1% FBS-supplemented medium in the upper chamber and 0.75 mL of 4% FBS-supplemented medium in the lower chamber. Cells were identified as migrating if they penetrated through the 8 µm pore inserts. In the treated conditions, 5 × 10^4^ cells were seeded in 0.5 mL of 0.1% FBS-supplemented medium with 20 mM BHB in the upper chamber and 0.75 mL of 4% FBS-supplemented medium in the lower chamber. The plate was incubated in humidified conditions, at 37 °C, and 5% CO_2_ for 8 h. Thereafter, media from the upper chambers were discarded, and a cotton swab was used to gently remove the non-migrating cells found on the top of the semipermeable membrane. Subsequently, the inserts holding the migrated cells on the lower semipermeable membrane surface were re-immersed in 100 μL medium combined with 100 μL CellTiter-Glo^®^. After 10 min, cells were lysed, and viability was detected via the CellTiter-Glo^®^ Luminescent Cell Viability assay (Promega Corporation, Madison, WI, USA) as described previously. BHB’s effect on cell migration was calculated and presented as a fold increase by comparing treated migrating cells with the second control.

### 4.7. Vascular Tube Formation Assay

Matrigel^®^ Matrix (Corning, Bedford, UK) was thawed to coat a 96-well plate, with 40 μL applied per well. The plate was placed on ice till an even bubble-free layer was formed. To allow Matrigel solidification, the plate was kept in a humidified incubator at 37 °C and 5% CO_2_ for 1 h. Subsequently, TeloHAEC cells were trypsinized and seeded on the coated plate at a density of 3 × 10^4^ cells/100 µL/well, with or without different BHB concentrations (10, 20, and 40 mM). After 6 h of incubation, photos of the formed vascular networks in different wells were captured using an Olympus inverted phase-contrast microscope (Olympus I×71, Olympus Corporation, Tokyo, Japan). The impact of BHB on TeloHAEC tube formation was evaluated by measuring the tubes’ total length, the number of branching points, and the number of formed loops in both control and BHB-treated wells. Those measurements were obtained using the online image analysis software Wimasis (https://www.wimasis.com/tube-formation-assay—access date 10 November 2022). The BHB effect on tube formation was calculated and presented in percentage (%) by comparing BHB-treated wells to the control wells, which are considered as 100%. The effect of various BHB concentrations on TeloHAEC cells’ viability at 6 h was evaluated using a CellTiter-Glo^®^ Luminescent Cell Viability assay (Promega Corporation, Madison, WI, USA), as previously described.

### 4.8. TeloHAEC Spheroids Sprouting Assay

The hanging drop method was used to form 3D spheroids from TeloHAEC cells. To prepare the cell suspension for four conditions, we used two 15 mL centrifuge tubes. In each, we added 190,000 cells into 2.5 mL EMB-2 medium and 625 μL 4% methylcellulose prepared in basal medium (Sigma-Aldrich, St. Louis, MO, USA). Next, 25 μL of the cell suspension was pipetted onto the inner side of a Petri dish cover. Finally, the Petri dish covers were flipped upside down, closing the Petri dishes and allowing the drops to hang inside. The dishes were placed in a humidified incubator at 37 °C and 5% CO_2_ for 24 h. A 4% FBS-supplemented methylcellulose was prepared to be added to the spheroid embedding mixture.

The next day, 20 mM BHB, 30 ng/mL VEGF, and a combination of both were prepared in basal medium and kept ready. Afterwards, TeloHAEC spheroids formed overnight were collected from each Petri dish cover into separate 15 mL centrifuge tubes using 1× PBS. Then they were centrifuged for 5 min at 150× *g*, with no break, and the supernatants were removed. Thereafter, the collagen mixture was prepared on ice by gently mixing 1800 μL of Collagen Type I, Rat Tail (Millipore, Burlington, MA, USA) with 180 μL of medium 199 (Sigma-Aldrich, St. Louis, MO, USA), and 41.4 μL of sterile ice-cold 1N NaOH to neutralize the collagen pH. To embed the spheroids, we worked on ice and began by adding 300 μL of the 4% FBS-supplemented methylcellulose prepared earlier to each TeloHAEC spheroid pallet, followed by 60 μL of basal medium or indicated treatment. Finally, 300 μL of the collagen mixture was added to each spheroid condition, mixed gently, and transferred into a 24-well plate. The plate was placed in a humidified incubator at 37 °C and 5% CO_2_ for 24 h, facilitating both collagen polymerization and spheroids sprouting. Photos were captured using the inverted microscope (Olympus I×71, Olympus Corporation, Tokyo, Japan), with 20× magnification. ImageJ (version 1.54g, built on the Java 1.8.0_345 platform) software was used to measure the total sprout length in 11 spheroids for each condition.

### 4.9. In Ovo Tumor Growth Assay

Fertilized Leghorn eggs, donated by Poultry Farm (Al Ain, UAE), were carefully wiped and placed in an R-com PRO50 digital egg incubator set at 37.5 °C and a humidity level of 55%. On embryonic day 3 (E3), a small hole was made at the narrow end of the eggshell using a 3 mL syringe with an 18 G needle to aspirate around 2 mL of the egg albumin. This procedure facilitates better exposure of the CAM during the experiment days. Subsequently, using fine scissors, a small round opening was meticulously cut in the eggshell directly above the CAM, then sealed with a semipermeable adhesive film (Suprasorb^®^ F - Lohmann & Rauscher International GmbH & Co. KG, Rengsdorf, Germany) and returned to the incubator till embryonic day 9 (E9). On E9, cancer cells, LNM35 and A549, were trypsinized, counted, and centrifuged. They were then suspended in equal amounts of Normal Saline (NS) and Matrigel^®^ Matrix (Corning, Bedford, UK) to achieve a cell count of 0.1 × 10^6^ cells/100 μL for LNM35 and 1 × 10^6^ cells/100 μL for A549. After that, the seal of each egg was carefully opened, and a small, autoclaved ring was inserted into each egg above the CAM area. Then, 100 μL of the cell suspension was inoculated into the ring of each egg, for a total of 14–15 eggs per condition.

On embryonic days 11, 13, and 15 (E11, E13, and E15), tumors formed were topically treated with NS for control, 100 mg/Kg BHB, 5 mg/Kg Gemcitabine, or a combination of both medicines prepared in NS. All steps were conducted under aseptic conditions. Ultimately, on embryonic day 17 (E17), the embryos were euthanized compassionately by topically applying 10 to 30 μL of Pentobarbitone Sodium (300 mg/mL, Jurox, Auckland, New Zealand). The tumors with the rings were cautiously extracted from the upper CAM tissue and washed with 1× PBS, each condition separately. Then, the tumors were cleaned carefully from the surrounding tissues and weighed to evaluate the effect of the drugs and their combination on the tumor growth. Data were presented by comparing the mean tumor weight of each group. The drugs’ toxic effect was evaluated at the end of the experiment by comparing the mean live embryos of each group. Chick embryos’ viability was tested by assessing the voluntary movements of the embryos and the pulsation and integrity of the blood vessels. This assay was performed as a randomly assigned, unblinded procedure, following the approved protocol by the animal ethics committee at the United Arab Emirates University. Additionally, the European Directive 2010/63/EU on the protection of animals used for scientific purposes stated that the experiments utilizing chicken embryos on or before embryonic day 18 (E18) do not require approval from the Institutional Animal Care and Use Committee (IACUC).

### 4.10. Bliss Independence Model

The Bliss independence model was applied to all combination data involving BHB and Gemcitabine to assess the type of their pharmacological interaction. This model estimates the expected combined effects of two drugs as the product of their individual effects, assuming that both drugs act independently. The expected combination effect was calculated using the following equation: E(a + b) = E(a) + E(b) − E(a) E(b), where E(a + b) is the expected effect, E(a) and E(b) are the respective effects of BHB and Gemcitabine at their specified concentrations. The excess over Bliss (EOB) score was calculated by subtracting the expected combination effect from the observed combination effect. The drug combinations were considered synergistic if EOB was higher than 0, antagonistic if EOB < 0, and additive if EOB = 0.

### 4.11. Statistical Analysis

Each experiment in this project was conducted at least three independent times, except for the CAM tumor growth assay. Data were presented as mean ± standard error of the mean (S.E.M). The statistical analysis was conducted using GraphPad Prism version 10 for Windows (GraphPad Software, San Diego, CA, USA). The unpaired *t*-test was applied to evaluate the difference between the two groups. The one-way ANOVA test, followed by Dunnett’s multiple comparison test, was applied to compare three or more groups to a control group. The one-way ANOVA test followed by Tukey’s multiple comparison test was applied in the combination experiments. Stars indicate the significance differences * *p* < 0.05, ** *p* < 0.01, *** *p* < 0.001, **** *p* < 0.0001.

## 5. Conclusions

In conclusion, our findings demonstrate that BHB inhibits endothelial cell-mediated angiogenesis in vitro and exerts significant anticancer activity against NSCLC cells both in vitro and in vivo using the CAM model. When combined with Gemcitabine, BHB further enhanced the inhibitory effects observed with Gemcitabine alone.

These findings provide preclinical evidence supporting further investigation of BHB alone or in combination with Gemcitabine in NSCLC. Extensive preclinical studies, incorporating a broader range of NSCLC cell lines and rodent xenografts, including patient-derived xenografts, are required to fully evaluate the translational potential of this therapeutic strategy. Moreover, the anti-angiogenic effects of BHB should be further validated in vivo, including assessment of CD31 expression in tumor xenografts. Collectively, these efforts will help define the clinical relevance and translational applicability of BHB in combination with standard-of-care therapies for the management of NSCLC.

## Data Availability

The original contributions presented in this study are included in the article. Further inquiries can be directed to the corresponding author.
